# Evaluation and molecular characterization of human adenovirus in drinking water supplies: viral integrity and viability assays

**DOI:** 10.1186/1743-422X-10-166

**Published:** 2013-05-28

**Authors:** Gislaine Fongaro, Mariana A do Nascimento, Caroline Rigotto, Giseli Ritterbusch, Alessandra D’ A da Silva, Paulo A Esteves, Célia R M Barardi

**Affiliations:** 1 Laboratório de Virologia Aplicada, Departamento de Microbiologia, Imunologia e Parasitologia, Universidade Federal de Santa Catarina, Florianópolis, 88040-900, Brazil; 2 Universidade Federal de Pelotas, Programa de Pós Graduação em Veterinária, Pelotas 96010-610, Brazil; 3 Embrapa Suínos e Aves, Concórdia 89700-000, Brazil

**Keywords:** HAdV, Water supply, Viral integrity and viability, ICC-RT-qPCR, Molecular characterization

## Abstract

**Background:**

Human adenoviruses (HAdVs) are the second-leading cause of childhood gastroenteritis worldwide. This virus is commonly found in environmental waters and is very resistant to water disinfection and environmental stressors, especially UV light inactivation. Molecular techniques, such as PCR-based methods (Polymerase Chain Reaction), are commonly used to detect and identify viral contamination in water, although PCR alone does not allow the discrimination between infectious and non-infectious viral particles. A combination of cell culture and PCR has allowed detection of infectious viruses that grow slowly or fail to produce cytopathic effects (CPE) in cell culture. This study aimed to assess the integrity and viability of human adenovirus (HAdV) in environmental water and evaluate circulating strains by molecular characterization in three sites of the water supply in Florianópolis, Santa Catarina Island, Brazil: Peri Lagoon water, spring source water, and water from the public water supply system.

**Methods:**

Water samples were collected, concentrated and HAdV quantified by real-time PCR. Viral integrity was evaluated by enzymatic assay (DNase I) and infectivity by plaque assay (PA) and integrated cell culture using transcribed mRNA (ICC-RT-qPCR). Samples containing particles of infectious HAdV were selected for sequencing and molecular characterization.

**Results:**

The analyzed sites contained 83, 66 and 58% undamaged HAdV particles (defined as those in which the genetic material is protected by the viral capsid) at Peri Lagoon, spring source water and public supply system water, respectively. Of these, 66% of the particles (by PA) and 75% (by ICC-RT-qPCR) HAdV were shown to be infectious, due to being undamaged in Peri Lagoon, 33% (by PA) and 58% (by ICC-RT-qPCR) in spring source water and 8% (by PA) and 25% (by ICC-RT-qPCR) in the public water supply system. ICC-RT-qPCR, a very sensitive and rapid technique, was able to detect as low as 1 × 10^2^ HAdV genome copies per milliliter of infectious viral particles in the environmental water samples. The molecular characterization studies indicated that HAdV-2 was the prevalent serotype.

**Conclusions:**

These results indicate a lack of proper public health measures. We suggest that HAdV can be efficiently used as a marker of environmental and drinking water contamination and ICC-RT-qPCR demonstrated greater sensitivity and speed of detection of infectious viral particles compared to PA.

## Background

Waterborne viral infection is one of the most important causes of human morbidity, and related diseases continue to have public health and socioeconomic implications worldwide. According to existing reports in the literature, there are hundreds of different types of human viruses present in human sewage, which, if improperly treated, can become a source of contamination in drinking and recreational water [1].

Human adenoviruses (HAdVs) are the second-leading cause of childhood gastroenteritis worldwide. This virus is commonly found in environmental waters and is very resistant to water disinfection and environmental stressors, especially UV light inactivation [2-4]. Along with other pathogens, HAdV is on the USEPA (United States Environmental Protection Agency) candidate contaminants list [5] as they are important human pathogens that can be transmitted by water consumption and water spray (aerosols).

Molecular techniques, such as PCR-based methods (Polymerase Chain Reaction), are commonly used to detect and identify viral contamination in water, particularly those viruses that do not multiply easily in cell culture [6]. The concentration of PCR inhibitors in environmental water samples and the ability of such techniques to detect naked nucleic acids from non-infectious pathogens may represent a limitation for the use of PCR as a detection tool. In addition, PCR alone does not allow the discrimination between infectious and non-infectious viral particles [7,8].

A combination of cell culture and PCR has allowed detection of infectious viruses that grow slowly or fail to produce cytopathic effects (CPE) in cell culture [6]. Integrated cell culture PCR (ICC-PCR) has the benefits of cell culture and PCR and attempts to compensate for several cell culture disadvantages, such as its time-consuming nature and limited detection sensitivity [9].

However, this method has a disadvantage in that detection of nucleic acids of inactivated viruses from environmental samples adsorbed onto cell receptors without cell infection can result in false positives [10]. Therefore, it is necessary to confirm infectious viruses by assaying infection of the permissive cells and subsequent transcription of viral mRNA. Thus, the detection of viral mRNA in cell culture indicates the presence of infectious viral particles [11].

Plaque assay is another method that can be used to infer the ability of viruses to infect and cause lysis in a cell monolayer [12]. Enzymatic assays can also be used to check the integrity of the viral capsid by using DNase or RNase nucleases. Genetic material that is not protected by a viral capsid will be degraded by these nucleases [6].

In this study, we aimed to assess the integrity and viability of human adenovirus (HAdV) detected in environmental water samples and also to use molecular characterization to evaluate circulating strains.

## Results

### Viral recovery assay

**Figure 1:**
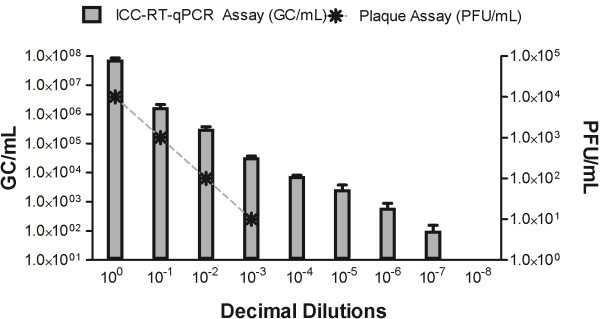
Viral recovery in water samples: Average viral recovery in water samples (through filtration, concentration, and a negatively charged membrane elution method) and viral detection test sensitivity, as measured by ICC-RT-qPCR assay and PA.

The efficiency of the viral recovery using the concentration method described by Katayama et al. (2002) [13] was measured. When analyzing the samples from all sites (1, 2, and 3), the recovery rate was approximately 10%, as evaluated with both PFU and GC units. The PA value (PFU/mL) equivalence, when compared to ICC-RT-qPCR values (GC/mL) immediately after the concentration of water samples and after a series of decimal dilutions (10^0^ to 10^-8^), is shown in Figure 1. The average equivalence of a PFU unit for GC was 3.4 logs, which means that 10^2^ PFU is equivalent to 10^5^ GC. This proportionality was confirmed via statistical testing (Linear Regression test) conducted with GraphPad Prism version 5.0 (USA).

The ICC-RT-qPCR assay was more sensitive than the PA in the detection of HAdV, demonstrating a detection limit of 1×10^2^ GC/mL (decimal dilution 10^-7^), while the PA had a detection limit of 1×10^1^ PFU/mL (decimal dilution 10^-3^) (Figure 1).

### Viral integrity and viability/infectivity in water samples

Twenty-five of 36 (69.5%) samples contained undamaged HAdV particles and 19/36 (52.7%) contained infectious HAdV particles. The rate (%) and the mean quantification of the undamaged and infectious HAdV particles, per site, are listed in Table 1.

**Table 1:** **Mean of the undamaged and infectious HAdV particles per site (*n*** **= 12)**

**HAdV**	**Lagoon water**	**Spring source water**	**Public supply system water**
Undamaged^*^	75.0 (9/12)	58.0(7/12)	66.6 (8/12)
Infectious^*^	75.0 (9/12)	25.0 (3/12)	58.0 (7/12)
Infectious in ICC-RT-qPCR^*^	75.0 (9/12)	25.0 (3/12)	58.0 (7/12)
Infectious per PA^*^	66.0 (8/12)	8.0 (1/12)	33.0 (4/12)
Undamaged^**^	1.65 × 10^6^	7.72 × 10^5^	1.24 × 10^6^
Infectious by ICC-RT-qPCR^**^	4.80 × 10^5^	3.69 × 10^3^	2.18 × 10^4^
Infectious by PA^***^	1.07 × 10^2^	3.33 × 10^0^	3.33 × 10^1^

* Rate (%). ** Mean (GC/L). *** Mean (PFU/L).

**Figure 2:**
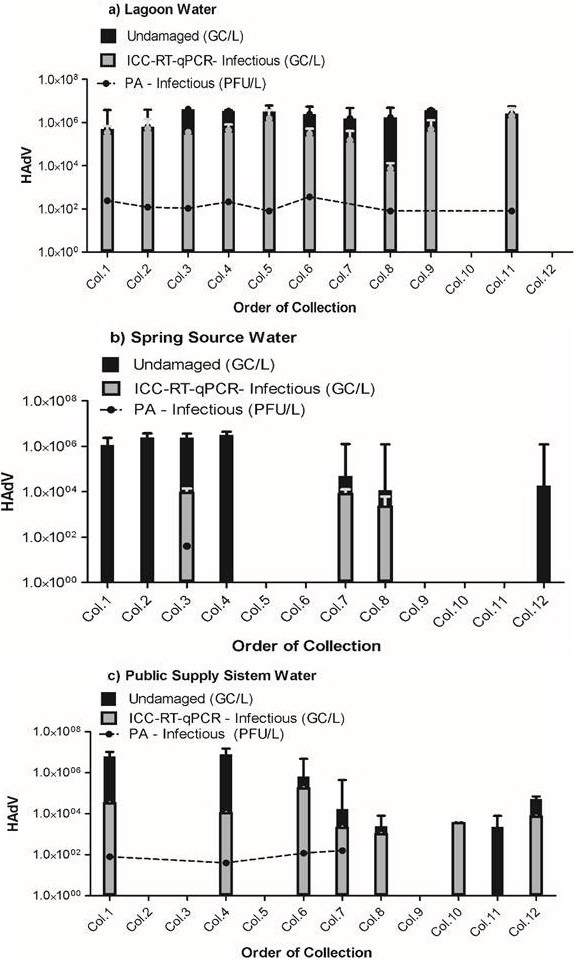
**Undamaged and infectious HAdV particle: Average number of undamaged and infectious HAdV particle detected in water samples: a) Lagoon water b) Spring source water, and c) Public supply system water.** In black bar: undamaged particles; in gray bar: infectious particle, measured by ICC-RT-qPCR assay, and in line: infectious particles, measured by (PA).

All positive sites (1, 2, and 3) samples contained undamaged and infectious viral particles. Not all samples with undamaged virus particles were shown to be infectious, but this difference was not statistically significant (*P* > 0.05, ANOVA in GraphPad). The ICC-RT-qPCR was more sensitive than the PA for detecting infectious particles in environmental water samples (Table 1 and Figure 2 (a, b, c)).

The results indicate that 10 of 12 lagoon water (site 1) samples contained undamaged viral particles. These all contained infectious particles detectable by ICC-RT-qPCR and 8 samples contained infectious particles detectable by PA. In addition, 7 of 12 source water (site 2) samples contained undamaged viral particles. Of these, only 3 samples contained infectious particles detectable by ICC-RT-qPCR and 1 sample contained particles detectable by PA. Lastly, 8 of 12 public supply system water (site 3) samples contained undamaged viral particles. Of these, 7 contained infectious particles detectable by ICC-RT-qPCR and 4 contained infectious particles detectable by PA. These results are presented in Figure 2 (a, b, and c).

### Sequencing and molecular characterization

**Figure 3:**
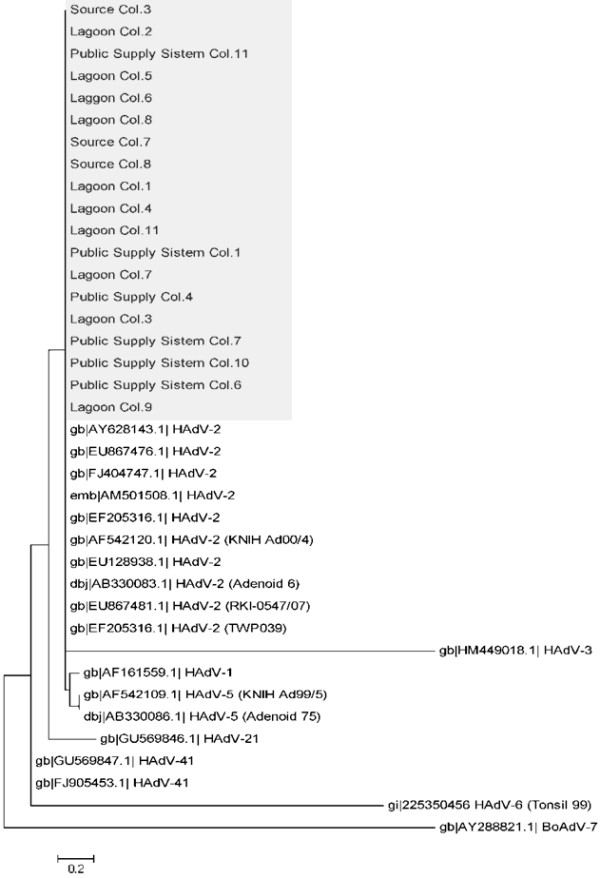
**Phylogenetic tree inferred by analysis of sequences of HAdV: Phylogenetic tree inferred by analysis of sequences corresponding to the region of HAdV obtained from the water lagoon, spring source and public supply system samples form from Florianopolis (underlined in gray).** The analyses were performed using maximum likelihood (based on Kimura 2 parameters with 500 replicates) with MEGA 5.0 software. The sequences used for comparison were accessed from the NCBI GenBank (not underlined). A sequence of bovine adenovirus (BovAdV-7, GenBank accession number AY288821) was used as an out group.

A total of 19 **s**amples (10 from lagoon water, 3 from spring source water, and 6 from public supply system water) that contained infectious or undamaged HAdV particles were sequenced for molecular characterization. The obtained sequences were compared with the ones already deposited in NCBI and presented 95-99% identical to sequences of HAdV serotype 2 of subgroup C. The resulting assembled phylogenetic tree is shown in Figure 3.

When compared, the amino acid sequences of 12 samples contained an amino acid substitution. This has been observed in the lagoon water samples (samples 1, 2, 3, 6, 7 and 11), the spring water samples (samples 3, 7 and 8) and the public water supply system samples (samples 1, 6 and 7). The effect of these substitutions was evaluated using the Phyre2 program, which indicated that the substitutions did not alter the secondary and tertiary structures of the HAdV hexon protein (data not shown).

## Discussion

Adenoviruses are among the most studied groups of potential viral indicators of water quality [1], This is due to the huge number of viral particles that are consistently found in environmental waters worldwide [4]. With that in mind, this study presents data on the integrity, infectivity and molecular characterization of HAdV in water samples (lagoon, spring source and public supply system) collected over a period of 1 year from Florianópolis, which is located in Southern Brazil.

In many countries, the water quality is evaluated according to bacteriological standards, even though bacterial contamination is not correlated with the presence of human enteric viruses [1]. The impact caused by water contamination of enteric viruses has been less studied than the impact caused by bacteria and protozoa. Due to the high cost of equipment and laboratory reagents, it can be difficult to detect viral agents in environmental samples, as they sometimes present in very low concentrations in the sampled material or because some viruses are not detectable in cell culture assays [14-17].

In this study, the filtration, concentration and elution method using a negatively charged membrane was applied to concentrate viruses from water samples. This method has been described as a useful tool to recover enteric viruses from environmental samples and allows the detection of such viruses by molecular methods [18-23].

As already reported in the literature, it is important to evaluate viral integrity (state of preservation of viral particles, with undamaged particles being defined as those in which the genetic material is protected by viral capsid) [6] and infectivity, (ability of the virus to replicate in permissive cells) [12], instead of only evaluating the presence or absence of the viral genome. Therefore, the processed samples were used to evaluate viral integrity (via enzymatic assay with DNase I) and infectivity (via PA and ICC-RT-qPCR-et) of HAdV present in the collected water samples.

The majority of samples evaluated in this study contained intact HAdV particles, but this result does not mean that those particles were infectious, as factors such as temperature, pH and UV radiation are known to cause conformational changes in the viral capsid, resulting in loss of infectivity [3]. On the other hand, HAdV has the ability to form aggregate particles in the water that protect the individual viral particles from inactivating factors [24].

All the samples containing particles of infectious HAdV detected by PA were also detected with ICC-RT-qPCR, but the reverse was not true. ICC-RT-qPCR demonstrated greater sensitivity and speed of detection of infectious viral particles compared to PA. ICC-RT-qPCR had a detection limit of 1×10^2^ GC/mL at 24 h. Specificity and sensitivity are also important aspects to consider, as the ICC-RT-qPCR relies on mRNA and thus avoids false negatives or positives [25,26].

The integrity of HAdV particles was positively correlated with their infectiousness, as evaluated by ICC-RT-qPCR, which corroborates other studies that have reported that enzymatic techniques are a valuable alternative when making inferences about potential viral infectivity, as they avoid laborious cell culture techniques and not all viruses can replicate in cell culture [27]. HAdV serotypes that are responsible for gastroenteritis are HAdV40 and 41 that are usually difficult to propagate in cell culture, so the use of enzyme tests can infer on viral infectivity, being a less laborious than techniques in cell culture [28].

Molecular and bioinformatics studies have both demonstrated higher efficiency in characterization, as in the discovery of new virus subtypes [29,30]. HAdV-2 is commonly found throughout the world. The virus subtype causes diseases that affect the upper respiratory tract, particularly in children [31,32]. The results of the HAdV characterization performed in the present study, by sequencing and molecular characterization, indicated the prevalence of HAdV, serotype subgroup C in the samples evaluated. The viruses that belong to HAdV subgroup C (C1-C2-C5-C6, and C7) are known to cause respiratory viral infections [33]. Our findings corroborate those of other studies that reported the prevalence of HAdV-2 in the aquatic environment and in stool samples [34-36]. Taken together, these studies and others indicate that HAdV-2 is one of the serotypes most commonly excreted by humans [37], suggesting that this virus is intermittently excreted in the feces of most individuals, even if they are asymptomatic [16].

Some substitutions in the HAdV amino acids sequences were observed and analyzed in this work. Other recent studies have observed substitutions of amino acids in respiratory HAdV, suggesting that the substitutions are related to respiratory-tissue viral tropism [38,39].

## Conclusion

Fecal pollution of drinking water is a primary health concern. The incidence of infectious HAdV in water samples indicates a contamination of these sources with human effluents. These results indicate a lack of proper public health measures, justifying the urgent necessity of adding methods for the detection, removal and inactivation of such viruses during the water treatment process. Furthermore, we suggest that HAdV can be efficiently used as a marker of environmental and drinking water contamination.

## Methods

### Water samples

Two liters of surface water samples were collected monthly for one year from three locations on Florianópolis, the capital of the state and island of Santa Catarina in Southern Brazil. A total of 36 samples were collected. Site 1 (Peri Lagoon), samples were collected from June 2010 to May 2011. This water source is used by the local water company plant and provides potable water for more than 100,000 inhabitants. Site 2 (spring source water - untreated) was collected from February 2011 to January 2012. Site 3 (public supply system water - spring source water chlorinated for human consumption) was collected from February 2011 to January 2012. The collective supply systems are controlled and regulated by the Ordinance of the Federal Ministry of Health of Brazil (MS 2914/2011) [40].

### Viral concentration method

Sample concentration was performed as described by Katayama et al. (2002) [13]. Briefly, this method consists of filtration, concentration, and elution of viral particles in water samples through a negatively-charged membrane. The final sample was approximately 10 mL before using a Centriprep Reconcentrated YM50® (Millipore), resulting in a final volume of approximately 5 mL, after centrifugation at 1,500 × g for 10 min at 4°C. The concentrated samples were stored at −80°C until further analysis.

### Genetic material extraction

The extraction of viral nucleic acid was performed using a commercial RTP DNA/RNA Virus® II Mini Kit (Invitek), according to the manufacturer's instructions. Nucleic acid was eluted in a 60 μL elution buffer and stored at −80°C until further analysis. A reverse transcriptase (RT) reaction was performed to generate cDNA from mRNA, using an RT enzyme and random primers (Sensiscript RT Kit – QIAGEN®).

### Real time quantitative PCR (qPCR)

Quantitative PCR was performed as described by Hernroth et al. (2002) [41]. All amplifications were done in a StepOne Plus® Real-Time PCR System (Applied Biosystems). Each sample was analyzed in triplicate. For each plate, four serial dilutions of standard were run in triplicate for each assay and the genome copies (gc) were measured. Ultra-pure water was used as the non-template control for each assay.

### Enzymatic assay

To infer the presence of undamaged viral particles, HAdV-positive samples, as detected by qPCR, were treated with DNase I, as described by Viancelli et al. (2011) [42]. To verify potential inhibitors of DNAse I present in the sample matrix, a known amount of previously inactivated HAdV-2 (1 h at 99°C and 30 min under UV irradiation) was added in concentrated samples (previously HAdV-2 negative) of all sites and in nuclease-free water (NFW), as a control. The reactions were performed using 1 U of DNAse (sufficient quantity to degrade 100% of DNA added), 1× buffer and 180 μL of sample or NFW and incubated for 15 min at room temperature, with the intention of degrading all free genetic material. Then, the enzyme was inactivated with EDTA 25 mM and incubated for 10 minutes at 65°C. These treated samples/NFW were then subjected to nucleic acid extraction and qPCR, as described previously.

### Cell line

For virus viability assays of environmental water samples, HAdV was propagated in a continuous line of A549 cells (permissive cells derived from human lung carcinoma cells, European Collection of Cell Cultures). These cells were kindly donated by Dr. Rosina Gironès from the University of Barcelona, Spain.

### Plaque assay (PA)

To infer the presence of infectious HAdV particles, water samples were previously treated with 10 U/mL penicillin, 10 μg/mL streptomycin and 0.025 μg/mL amphotericin B. The treated samples were inoculated (0.25 mL) at a non-cytotoxic dilution, in triplicate, in A549 cells for the plaque assay, as described by Cromeans et al. (2008) [43] and Rigotto et al. (2011) [44]. The cells were incubated for 1 h at 37°C with rotation every 15 min. The cells were then removed and overlaid with 0.6% Bacto-agar (previously melted) in high glucose DMEM 2X (high-glucose Dulbecco’s Modified Eagle’s Medium) containing 4% FBS, 0.1 mM sodium pyruvate, 10 U/mL penicillin, 10 μg/mL streptomycin and 26 mM MgCl_2_. The samples were then incubated at 37 °C for 7 days. Then, the agar overlay was removed, and the cells were stained with 20% Gram’s crystal violet and the plaques counted macroscopically.

### Integrated cell culture-RT-qPCR assay (ICC-RT-qPCR)

To quantify the number of infectious HAdV particles present in the water samples, an ICC-RT-qPCR assay (integrated cell culture – preceded by reverse transcriptase and qPCR) was conducted. The protocol was adapted from Rigotto et al. (2005) [45] and Ko et al. (2003) [11] and aims to access viral mRNA following viral replication in cells.

Water samples, in a non-cytotoxic dilution, were inoculated in triplicate in A549 cells for the ICC-RT-qPCR assay. After 1 h of incubation at 37°C with rotation every 15 min, the inoculum was removed and the cell layers were overlaid with high-glucose Dulbecco’s Modified Eagle’s Medium (DMEM) before being incubated at 37°C for 24 h. After incubation as described previously, the supernatant was recovered and 0.2 mL was used for genetic material extraction, as described above. Immediately after the extraction of the total nucleic acids, enzymatic treatment, with DNase I, was conducted in order to degrade the DNA in the sample tested. Then a reverse transcriptase reaction (RT) was used to generate cDNA from viral mRNA. The quantification of infectious particles of HAdV was performed with qPCR, as described above.

### Viral recovery assay

For each site analyzed, to determine the efficiency of the viral recovery by the concentration method, a known concentration of HAdV was spiked into 2 L of each water matrix (previously negative for HAdV by qPCR). The samples were concentrated, and the viral recovery was assessed by plaque assay (PA) and by integrated cell culture-RT-qPCR (ICC-RT-qPCR), as described below. To determine the equivalence of the PA viral quantification to the ICC-RT-qPCR value, and also to confirm the ability of these techniques to detect infectious HAdV in each water matrix, decimal dilutions (10^0^ to 10^-8^) of concentrated seeded water samples were created and analyzed.

### Sequencing and molecular characterization

Samples containing particles of infectious HAdV were selected for sequencing and molecular characterization. The primers described by Allard et al. (1992) [46], directed to a region of the viral genome coding for the capsid *hexon* protein (18858–19158 bp position in HAdV genome) common to all HAdVs, were used for PCR, generating a product of approximately 300 bp. The amplicons were purified with ExoSAP-IT (GE Healthcare UK Ltd, Buckinghamshire, UK). Ultra-pure water was used as the non-template control for each assay.

Sequencing was carried out in a 3130 Genetic Analyzer with the Big Dye Terminator v3.1 Cycle Sequencing Kit (Applied Biosystems) and following the manufacturer’s protocol. Each product was sequenced four times in both directions. The quality of DNA sequences was checked and overlapping fragments were assembled using the BioEdit package 7.0.5 [47], and ContigExpress (InforMax, Inc.). After the alignment, the sequences were compared with the ones deposited in GenBank using the BLAST tool [48] and the molecular characterization was conducted with MEGA 5.0 software [49]. The homology analyses (evolutionary history) were inferred by using the Maximum Likelihood method based on the Kimura 2 - parameter model and calculated using pairwise deletion. Bootstrap was resampled as a test of phylogeny using 500 replications [50].

### Statistical analyses

The statistical analyses were performed using GraphPad Prism version 5.0 (USA). A Pearson correlation and linear regression test, ANOVA test and Student’s t test were performed (*P* < 0.05).

## Consent

Written informed consent was obtained from the patient for publication of this report and any accompanying images.

## Competing interests

The authors declare that they have no competing interests.

## Authors’ contributions

GF, MAN and CR design and carried out the research, GR, ADS and PAE contributed in the sequence analysis, and GF and CRMB wrote the paper. All authors read and approved the final manuscript.

## References

[B1] Fong TT Lipp EK Enteric viruses of human and animals in aquatic environments: health risks, detection, and potential water quality assessment tools Microbiol Mol Biol Rev 2005 69 357 371 10.1128/MMBR.69.2.357-371.2005 15944460 PMC1197419

[B2] Crabtree KD Gerba CP Rose JB Haas CN Waterborne adenovirus: A risk assessment Water Sci Technol 1997 35 1 6

[B3] Thurston-Enriquez JA Haas CN Jacangelo J Riley K Gerba CP Inactivation of feline calicivirus and adenovirus type 40 by UV radiation Appl Environ Microbiol 2003 69 577 582 10.1128/AEM.69.1.577-582.2003 12514044 PMC152434

[B4] Gerba CP Gramos DM Nwachuku N Comparative inativation of enteroviruses and adenovirus 2 by UV light Appl Environ Microbiol 2002 68 5167 5169 10.1128/AEM.68.10.5167-5169.2002 12324370 PMC126408

[B5] USEPA – United States Environmental Protection Agency Contaminant candidate list 3 2009 – CCL*in* http://water.epa.gov/scitech/drinkingwater/dws/ccl/ccl3.cfm21937977

[B6] Girones R Ferrús MA Alonso JL Rodriguez-Manzano J Calgua B Corrêa AA Hundesa A Carratala A Bofill-Mas S Molecular detection of pathogens in water - The pros and cons of molecular techniques Water Res 2010 44 4325 4339 10.1016/j.watres.2010.06.030 20619868

[B7] Choi S Chiang SC Real-time PCR quantification of human adenoviruses in urban rivers indicates genome prevalence but low infectivity Appl Environ Microbiol 2005 71 7426 7533 10.1128/AEM.71.11.7426-7433.2005 16269784 PMC1287606

[B8] Hamza IA Jurzik L Überla K Wilhelm M Evaluation of pepper mild mottle virus, human picobirnavirus and Torque teno virus as indicators of fecal contamination in river water Water Res 2011 45 1358 1368 10.1016/j.watres.2010.10.021 21074236

[B9] Nuanualsuwan S Cliver DO Pretreatment to avoid positive RT-PCR results with inactivated viruses Jour 2002 104 217 225 10.1016/S0166-0934(02)00089-7 12088831

[B10] Sobsey MD Battigelli DA Shin GA Newland S RT-PCR amplification detects inactivated viruses in water and wastewater Water Sci Technol 1988 38 91 94

[B11] Ko G Cromeans TL Sobsey MD Detection of infectious adenovirus in cell culture by mRNA reverse transcription-PCR Appl Environ Microbiol 2003 69 7377 7384 10.1128/AEM.69.12.7377-7384.2003 14660388 PMC309946

[B12] Herzog P Drosten C Muller MA Plaque assay for human coronavirus NL63 using human colon carcinoma cells Virol J 2008 5 138 10.1186/1743-422X-5-138 19014487 PMC2603006

[B13] Katayama H Shimasaki A Ohgaki S Development of a virus concentration method and its application to detection of enterovirus and Norwalk virus from coastal seawater Appl Environ Microbiol 2002 68 1033 1039 10.1128/AEM.68.3.1033-1039.2002 11872447 PMC123733

[B14] Calgua B Mengewein A Grünert A Bofill-Mar S Clemente-Casares P Hundesa A Wyn-Jones AP López-Pila JM Girones R Development and application of a one-step low cost procedure to concentrate viruses from seawater samples J Virol Methods 2008 153 79 83 10.1016/j.jviromet.2008.08.003 18765255

[B15] Wyn-Jones AP Sellwood J Enteric viruses in the aquatic environment J Appl Microbiol 2001 91 945 962 10.1046/j.1365-2672.2001.01470.x 11851802

[B16] Wyn-Jones AP Carducci A Cook N D’agostino M Divizia M Fleischer J Gantzer C Gawler A Girones R Holler C Husman AMR Kay D Kozyra I López-Pila J Muscillo M Nascimento MSJ Papageorgiou G Rutjes S Sellwood J Szewzyk R Wyer M Surveillance of adenoviruses and noroviruses in European recreational waters Water Res 2011 45 1025 1038 10.1016/j.watres.2010.10.015 21093010 PMC7112131

[B17] Bofill-Mas S Albinana-Gimenez N Clemente-Casares P Hundesa A Rodriguez-Manzano J Allard A Calvo M Girones R Quantification and stability of human adenoviruses and polyomavirus JCPyV in wastewater matrices Appl Environ Microbiol 2006 72 7894 7896 10.1128/AEM.00965-06 17028225 PMC1694247

[B18] Fumian TM Leite JPG Castello AA Gaggero A Caillou MSL Miagostovich MP Detection of rotavirus A in sewage samples using multiplex qPCR and an evaluation of the ultracentrifugation and adsorption-elution methods for virus concentration J Virol Methods 2010 170 42 46 10.1016/j.jviromet.2010.08.017 20804786

[B19] Haramoto E Kitajima M Katayama H Ohgaki S Real-time PCR detection of adenoviruses, polyomaviruses, and torque teno viruses in river water in Japan Water Res 2006 44 1747 1752 19969322 10.1016/j.watres.2009.11.043

[B20] Miagostovich MP Ferreira FFM Guimarães FR Fumian TM Diniz-Mendes L Luz SLB Silva LA Leite JPG Molecular detection and characterization of gastroenteritis viruses occurring naturally in the stream waters of Manaus, Central Amazônia Brazil Appl Environ Microbiol 2008 74 375 382 10.1128/AEM.00944-07 18065620 PMC2223260

[B21] Rigotto C Victoria M Moresco V Kolesnikovas CKM Correa AA Souza DSM Miagostovich M Simões CM Barardi CR Assessment of adenovirus, hepatitis A virus and rotavirus presence in environmental samples in Florianópolis South Brazil J Appl Microbiol 2010 109 1979 1987 10.1111/j.1365-2672.2010.04827.x 20698910

[B22] Victoria M Guimarães F Fumian T Ferreira F Vieira C Leite JP Miagostovich M Evaluation of an adsorption-elution method for detection of astrovirus and norovirus in environmental waters J Virol Methods 2009 156 73 76 10.1016/j.jviromet.2008.11.003 19056426

[B23] Villar LM de Paula VS Diniz-Mendes L Guimarães FR Ferreira FF Shubo TC Miagostovich MP Lampe E Gaspar AM Molecular detection of hepatitis A virus in urban sewage in Rio de Janeiro Brazil Lett Appl Microbiol 2007 45 168 173 10.1111/j.1472-765X.2007.02164.x 17651213

[B24] Bosch A Human enteric viruses in the water environment: a minireview Int Microbiol 1998 1 191 196 10943359

[B25] Ko G Cromeans TL Sobsey MD UV inactivation of adenovirus type 41 measured by cell culture mRNA RT-PCR Water Res 2005 39 3643 3649 10.1016/j.watres.2005.06.013 16046229

[B26] Hong-Xia M Lin Z Yuan Z Rapid quantification of infectious enterovirus from surface water in Bohai Bay, China using an integrated cell culture-qPCR assay Mar Pollut Bull 2011 62 2047 2054 10.1016/j.marpolbul.2011.07.024 21889173

[B27] Rodríguez RA Pepper IL Yerba CP Application of PCR-based methods to assess the infectivity of enteric viruses in environmental samples Appl Environ Microbiol 2009 75 297 307 10.1128/AEM.01150-08 19011062 PMC2620694

[B28] Kovac K Bouwknegt M Diez-Valcarce M Raspor P Hernández M Rodríguez-Lázaro D Evaluation of high hydrostatic pressure effect on human adenovirus using molecular methods and cell culture Int J Food Microbiol 2012 157 368 374 10.1016/j.ijfoodmicro.2012.06.006 22732528

[B29] Liu EB Ferreyra L Fischer SL Pavan JV Nates SV Hudson NR Tirado D Dyer DW Chodosh J Seto D Jones MS Genetic analysis of a novel human adenovirus with a serologically unique hexon and a recombinant fiber gene PLoS One 2011 6 24491 24501 10.1371/journal.pone.0024491 PMC3168504 21915339

[B30] Ishiko H Aoki K Spread of epidemic keratoconjunctivitis due to a novel serotype of human adenovirus in Japan J Clin Microbiol 2009 47 2678 2679 10.1128/JCM.r00313-09 19644130 PMC2725644

[B31] Foy HM Evans AS, Kaslow RA Adenovirus Viral infections of humanas: epidemiology and control 1997 New York: Plenum Medical Book Company 119 138 ISBN 4

[B32] Videla C Carballal G Misirlian A Aguila M Acute lower respiratory infections due to respiratory syncytial virus and adenovirus among hospitalised children from Argentina J Clin Virol 1998 10 17 23 10.1016/S0928-0197(98)00017-8 9645999

[B33] Wold WSM Horwitz MS Knipe DM, Howley PM Adenoviruses Fields virology 2007 Philadelphia: Lippincott Williams & Wilkins 2395 2436

[B34] Lee IJ Lee GC Chung JY Han TH Lee YK Kim MS Lee CH Detection and molecular characterization of adenoviruses in Korean children hospitalized with acute gastroenteritis Microbiol Immunol 2012 56 523 528 10.1111/j.1348-0421.2012.00469.x 22530970

[B35] Barrero PR Valinotto LE Tittarelli E Mistchenko AS Molecular typing of adenoviruses in pediatric respiratory infections in Buenos Aires, Argentina (1999–2010) J Clin Virol 2012 53 145 150 10.1016/j.jcv.2011.11.001 22138300

[B36] Moresco V Viancelli A Nascimento MA Souza DMS Ramos APD Garcia LAT Simões CMO Barardi CRM Microbiological and physicochemical analysis of the coastal waters of southern Brazil Mar Pollut Bull 2012 64 40 48 10.1016/j.marpolbul.2011.10.026 22104718

[B37] Mena KD Gerba CP Waterborne Adenovirus Rev Environ Contam Toxicol 2009 198 133 167 19253037 10.1007/978-0-387-09647-6_4

[B38] Kaneko H Iida T Ishiko H Analysis of the complete genome sequence of epidemic keratoconjunctivitis related human adenovirus type 8, 19, 37 and a novel serotype J Gen Virol 2009 90 1471 1476 10.1099/vir.0.009225-0 19264666

[B39] Tohma K Bayasgalan N Suzuki A Darma B Oshitani H Nymadawa P Detection and serotyping of human adenoviruses from patients with influenza-like illness in mongolia Jpn J Infect Dis 2012 65 289 294 10.7883/yoken.65.289 22814149

[B40] MS 2914/2011 Portaria MS Nº 2.914 12th December 2011 in http://www.agenciapcj.org.br/novo/images/stories/portaria-ms-2914.pdf

[B41] Hernroth BE Conden-Hansson AC Rehnstan-Holm AS Girones R Allard AK Environmental factors influencing human viral pathogens and their potential indicator organisms in the blue mussel, *mytilus edulis:* the first Scandinavian report Appl Environ Microbiol 2002 68 4523 4533 10.1128/AEM.68.9.4523-4533.2002 12200309 PMC124092

[B42] Viancelli A Garcia LAT Kunz A Steinmetz R Esteves PA Barardi CRM Detection of circoviruses and porcine adenoviruses in water samples collected from swine manure treatment systems Res Vet Sci 2012 93 538 543 10.1016/j.rvsc.2011.07.022 21872287

[B43] Cromeans TL Lu XY Erdman DD Humphrey CD Hill VR Development of plaque assays for adenoviruses 40 and 41 J Virol Methods 2008 151 140 145 10.1016/j.jviromet.2008.03.007 18440077

[B44] Rigotto C Hanley K Rochelle PA De Leon R Barardi CRM Yates MV Survival of adenovirus types 2 and 41 in surface and ground waters measured by a plaque assay Environ Sci Technol 2011 45 4145 4150 10.1021/es103922r 21480609

[B45] Rigotto C Sincero TC Simões CM Barardi CR Detection of adenoviruses in shellfish by means of conventional-PCR, nested-PCR, and integrated cell culture PCR (ICC ⁄ PCR) Water Res 2005 39 297 304 10.1016/j.watres.2004.10.005 15644238

[B46] Allard A Albinsson B Wadell G Detection of adenoviruses in stools from healthy persons and patients with diarrhea by two-step polymerase chain reaction J Med Virol 1992 37 149 157 1629713 10.1002/jmv.1890370214

[B47] Hall TA BioEdit: a user-friendly biological sequence alignment editor and analysis program for Windows 95/98/NT Nucleic Acids Symp Ser 1999 41 95 98

[B48] Altschul SF Madden TL Schäffer AA Zhang J Zhang Z Miller W Lipman DJ Gapped BLAST and PSI-BLAST: a new generation of protein database search programs Nucleic Acids Res 1997 25 3389 3402 10.1093/nar/25.17.3389 9254694 PMC146917

[B49] Tamura K Peterson D Peterson N Stecher G Nei M Kumar S MEGA5: molecular evolutionary genetics analysis using maximum likelihood, evolutionary distance, and maximum parsimony methods Mol Biol Evol 2011 28 2731 2739 10.1093/molbev/msr121 21546353 PMC3203626

[B50] Efron B Halloran E Holmes S Bootstrap confidence levels for phylogenetic trees PNAS 1996 93 13429 13434 10.1073/pnas.93.23.13429 8917608 PMC24110

